# GENERIC AND TAILORED INTERVENTIONS FOR COMMUNITY OLDER ADULTS WITH CHRONIC COGNITIVE COMMUNICATION DISORDERS

**DOI:** 10.1093/geroni/igac059.140

**Published:** 2022-12-20

**Authors:** Ladan Ghazi Saidi

**Affiliations:** Universoty of Nebraska at Kearney, Kearney, Nebraska, United States

## Abstract

Many Community-Dwelling Older Adults (CDOA) live with debilitating chronic cognitive and communication disorders including Aphasia, Mild Cognitive Impairment, and Traumatic Brain Injury, and no longer receive healthcare services. This portion of the symposium will discuss why engaging CDOA and their caregivers with developing and selecting interventions for their cognitive communication disorders is important. Further, we discuss a community-based approach to make generic as well as tailored speech-language pathology services available to CDOA free of charge. The process of developing and using a multi-stakeholder advisory board of clinicians, researchers, CDOA, as well as a diverse advisory board of caregivers will be described, and challenges will be discussed. Further, we will present and discuss some relevant data.

